# Quality of randomized controlled trials reporting in the treatment of melasma conducted in china

**DOI:** 10.1186/s13063-015-0677-2

**Published:** 2015-04-12

**Authors:** Zhiwei Chen, Yuchi Chen, Jingchun Zeng, Yang Wang, Teng Ye, Qiaochu Zhou, Xiaojing Du, Wenting Su, Zhishan Ding

**Affiliations:** Department of Dermatology, Wenzhou Hospital of Integrated Traditional Chinese and Western Medicine, Wenzhou Children’s Hospital, Wenzhou, 325000 Zhejiang Province China; College of Life Sciences, Zhejiang Chinese Medical University, Hangzhou, 310053 Zhejiang Province China; Guangzhou University of Chinese Medicine, Guangzhou, 510405 China; Zhejiang Chinese Medical University, Hangzhou, 310053 Zhejiang Province China

**Keywords:** CONSORT statement, Melasma, Quality of reporting, Randomized controlled trial

## Abstract

**Background:**

There is no existing report on the quality of randomized controlled trials (RCTs) of melasma treatment currently conducted in China. This study aims to assess the quality of RCT- reporting in the treatment of melasma conducted in China.

**Methods:**

Several databases were searched from their inception through to August 2014. In order to rate the report quality, we scored 1 for the item if it was reported in CONSORT 2010 and 0 for the item if it was not definitely stated or was not clear. For overall quality score (OQS), 13 items were scored and calculated with a range of 0 to 13. Five items (‘randomization’, ‘allocation concealment’, ‘blinding’, ‘baseline characteristics’ and ‘intention-to-treat (ITT) analysis’) were scored and a key methodological index score (MIS) was calculated with a range of 0 to 5 for each trial.

**Results:**

A total of 246 relevant RCTs were included in the final analysis. The median OQS was 7, with a minimum of 4 and a maximum of 11. Some items’ information was insufficient, especially in the categories of ‘trial design’, ‘sample size’, ‘recruitment’ and ‘ancillary analyses’ with a positive rate of less than 20%. The median MIS was 1 with a minimum of 0 and a maximum of 3. Some items’ reporting was poor, especially in the categories of ‘randomization’, ‘allocation concealment and implementation’, ‘blinding’ and ‘ITT analysis’ with a positive rate of less than 10%. The mean OQS increased by about 0.52 for manuscripts published in the period of 5-year increments (95% CI: 0.32 to 0.72; *P* < 0.001). With regard to the MIS, no variable was statistically significant in the ordinal regression model.

**Conclusion:**

The reporting quality of RCTs in the treatment of melasma conducted in China is not satisfactory especially in key methodological items. Reporting of RCTs in this field should meet and keep up with the standards of the CONSORT statement.

**Electronic supplementary material:**

The online version of this article (doi:10.1186/s13063-015-0677-2) contains supplementary material, which is available to authorized users.

## Background

Melasma is an acquired increased pigmentation of the skin characterized by symmetrical and confluent grey-brown patches mostly on the areas of the face exposed to the sun, such as over the cheek bones, forehead, and chin [1]. Although the prevalence of melasma has not been investigated in most countries, melasma accounts for about 4 to 10% of new cases in dermatology hospitals [2] and is most common in Hispanics, darker-skinned patients, and Asians [3]. In China, the overall prevalence of melasma is 3.23 to 13.61%, being 0.36 to 8.33% for males and 4.65 to 17.98% for females [4,5].

The causes of melasma are still unclear and the treatment is often unsatisfactory. In general, available therapies consist of preparations applied to the skin (for example, sunscreens that block ultraviolet light, topical steroids, topical retinoids, azelaic acid and kojic acid) and, recently, laser therapy. In China, internal Chinese medicine, acupuncture, moxibustion and combined therapies are also common in the treatment of melasma. To find the more efficient therapies, more and more randomized controlled trials (RCTs) in melasma have recently emerged. However, it is still unknown what the reporting quality of RCTs is in melasma studies conducted in China. RCTs, as a ‘gold standard’ of evidence-based clinical practice, are generally considered to have the highest level of credibility in determining efficacy of a new treatment [6]. A well-conducted RCT reporting on melasma is an effective tool to scientifically present evidence for duplication, experts’ evaluations, peer review decisions and systematic reviews. Biased results of RCT reports with poor quality will mislead treatment decisions and the formulation of a national public health strategy [7,8].

The aim of this study was to assess the quality of RCT-reporting in the treatment of melasma conducted in China. Furthermore, we will be able to advise on improvements in the reporting quality in this field after we know the status.

## Methods

### Search strategy

The following databases were searched from their inception through to August 2014: the Cochrane Central Register of Controlled Trials (CENTRAL), MEDLINE, EMBASE, CINAHL, AMED, the Chinese Biomedical Database, China National Knowledge Infrastructure, Wanfang databases and VIP Information. Other resources were: reference lists of eligible studies and previous systematic reviews were also reviewed to identify further eligible studies. The following search terms were used in Chinese and English: melasma, melanosis, chloasma, randomized trials, RCT, China, and so on.

### Inclusion and exclusion criteria

Types of studies: RCTs that assessed the effect of any intervention for melasma conducted in China. However, quasi-randomized trials, non-randomized, cross-over RCTs, case reports, and case-control were excluded. All RCTs had to be performed in China and mainly by Chinese researchers.

Types of interventions: we considered all types of interventions; this included studies that compared at least one active treatment with a control, which may be a placebo or an alternative intervention.

Types of participants: people of all age groups and ethnic backgrounds who had a clinical diagnosis of melasma made by a physician.

### Assessment of reporting quality

#### Rating of overall reporting quality

In order to rate the report quality, we scored 1 for the item if it was reported in CONSORT 2010 [9,10] and 0 for the item if it was not definitely stated or was not clear. For overall quality score (OQS), 13 items were scored and calculated with a range of 0 to 13 (Table 1) [8,10,11].

**Table 1:** **Overall quality of reporting rating using items from the Consolidated Standards for Reporting Trials (CONSORT) statement (n = 246)**

**Item**	**Criteria**	**Description**	**Number of positive trials**	**%**	**Cohen’s** ***к*** **coefficient**	**95% CI**
1	‘Randomized’ in the title or abstract	Study identified as a randomized controlled in the title or abstract	226	92	1	1
2	Background	Adequate description of the scientific background and explanation of rationale	70	29	0.72	0.63 to 0.79
3	Trial design	Description of trial design (such as parallel, factorial) including allocation ratio	5	2	0.69	0.56 to 0.75
4	Participants	Description of the eligibility criteria for participants	194	79	0.93	0.85 to 0.99
5	Interventions	Details of the interventions intended for each group	244	99	0.75	0.66 to 0.88
6	Outcomes	Definition of primary (and secondary when appropriate) outcome measures	201	82	0.83	0.74 to 0.98
7	Sample size	Description of sample size calculation	0	0	0.76	0.68 to 0.97
12	Statistical methods	Description of the statistical methods used to compare groups for primary outcomes, subgroup analyses, or adjusted analyses	241	98	0.81	0.72 to 0.93
13	Flow chart	Details on the flow of participants through each stage of the trials (number of patients randomly assigned, receiving intended treatment, completing the protocol and analyzed)	242	98	0.95	0.92 to 0.98
14	Recruitment	Dates defining the periods of recruitment and follow-up	31	13	0.69	0.60 to 0.78
17	Outcomes and estimation	For each primary and secondary outcome, a summary of results for each group is given, and the estimated effect size and its precision (for example, 95% CI)	244	99	0.82	0.70 to 0.96
18	Ancillary analyses	Clear statement of whether subgroup/adjusted analyses were prespecified or exploratory	19	8	0.73	0.65 to 0.83
19	Harms	Description of all important adverse events in each group	113	46	0.78	0.73 to 0.86

#### Rating of key methodological items

Five key methodological categories of ‘randomization’, ‘allocation concealment’, ‘blinding’, ‘baseline characteristics’ and ‘intention-to-treat (ITT) analysis’ have been assessed separately because they relate to potential sources of bias [12-14]. We then developed eight ‘yes’/‘no’ items (Table 2), wording so that emphasis was placed on quality of reporting rather than adequacy of trial design. Each item was scored 1 if the method was appropriate and 0 if inappropriate or if the reporting was unclear. Five items were scored and a key methodological index score (MIS) were calculated with range of 0 to 5 for each trial.

**Table 2:** **Reporting quality of key methodological items (n = 246)**

**Item**	**Criteria**	**Description**	**Number of positive trials**	**%**	**Cohen’s** ***к*** **coefficient**	**95% CI**
8	Randomization	Description of the method used to generate the random sequence	41	17	0.85	0.76 to 0.95
9 and 10	Allocation concealment and implementation	Description of the method used to implement the random allocation sequence assuring the concealment until interventions are assigned	1	0	0.75	0.65 to 0.88
11	Blinding	Whether or not participants, those administering the interventions, or those assessing the outcomes were blinded to group assignment	6	2	0.78	0.72 to 0.87
15	Baseline data	An outline of baseline demographic and clinical characteristics of each group	147	60	0.82	0.78 to 0.90
16	Intent-to-treat analysis	Number of participants in each group included in each analysis and whether it was done by ‘intention-to-treat’	5	2	0.86	0.78 to 0.98

### Data extraction and analysis

Each article was reviewed by two independent investigators (YW and TY). Two investigators, blinded to each other’s ratings, completed the rating form independently. Statistics used a modified CONSORT checklist with 18 items (Tables 1 and 2). Cohen’s *к*-statistic was calculated to assess agreement between two assessors. Agreement was judged as poor if *к ≤* 0.20; fair if 0.20 lower than *к ≤* 0.40; moderate if 0.40 lower than *к ≤* 0.60; substantial if 0.60 lower than *к ≤* 0.80; good if *к* higher than 0.80; and perfect if *к =* 1 [8,10]. Discrepancies were reviewed in detail and subsequently settled by consensus.

To explore factors associated with OQS, a multiple linear regression model was performed only if variables in the univariate models were significant at *P* ≤ 0.10. In the final multivariate model, variables were significant predictors at *P* ≤ 0.05. As the OQS variable was in a normal distribution, we believed that employing a multiple linear regression model is enough to obtain an approximate *P*-value with the clarity of the coefficient interpretation (not the collinearity relationship between different independent variables from the result of the collinearity diagnosis) and the large number of cases (246 cases).

As the outcome variable, MIS could be considered as a count and the ordinal regression model was used to select factors associated with MIS. The independent factors for these models were ‘year of publication’, ‘funding’, ‘type of interventions’ and ‘type of journals’. Descriptive statistical analysis, linear and ordinal regression analysis were performed using SPSS version 20.0 (SPSS, Chicago, IL, USA). Analyses of Cohen’s *к*-statistics were performed using the SAS software, version 9.3 (SAS Institute, Inc., Cary, NC, USA). Database of RCTs reporting in the treatment of melasma conducted in China are provided in Additional file 1.

## Results

**Figure 1:**
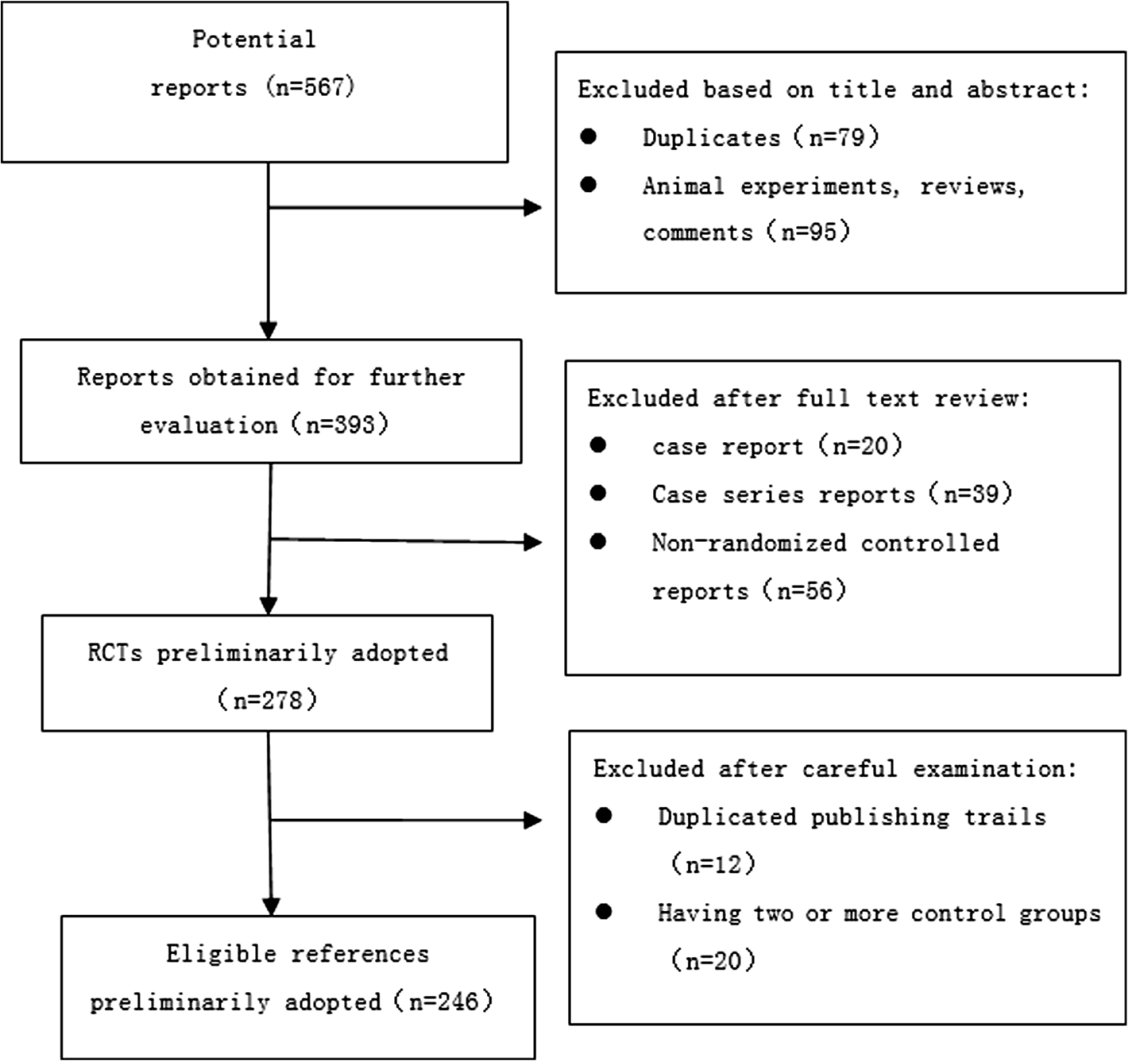
Flowchart of the article selection process.

The RCTs selection process is outlined in Figure 1. The researchers applied the search method to find 567 reports related to the topic. After carefully selecting, a total of 246 relevant RCTs were included in the final analysis (Figure 1).

### Characteristics of included trials

The number of RCT reports on the treatment of melasma conducted in China is increasing. In the periods of ‘before 1999’, ‘2000 to 2004’, ‘2005 to 2009’ and ‘2010 to 2014’, 7 articles (2.8%), 13 articles (5.3%), 95 articles (38.6%) and 131 articles (53.3%), respectively emerged. For the journal type, 67 (27.2%), 45 (18.3%) and 134 (54.5%) were published in general medical journals, specialist medical journals and traditional and alternative medical journals, respectively. In terms of financial sources, 29 articles (11.8%) reported their funding sources, most of which were obtained from provincial and municipal sources. In terms of choice of interventions, the largest number of interventions were comprised of internal Chinese medicine (90 reports; 36.6%) followed by combined medicine (40 reports; 16.3%), drugs (25 reports; 10.2%), acupuncture and moxibustion (23 reports; 9.3%), ultrasonic therapy (6 reports; 2.4%), laser therapy (5 reports; 2.0%) and others (57 reports; 23.2%).

### Quality of reporting

#### Rating of overall reporting quality

The overall quality of reporting rating using items from the CONSORT statement was listed in Table 1. The overall level of reporting was not good; the median OQS was 7, with a minimum of 4 and a maximum of 11. Some items’ information was insufficient, especially in the items of ‘trial design’, ‘sample size’, ‘recruitment’ and ‘ancillary analyses’ with a positive rate of less than 20%. A substantial agreement was observed for items 2, 3, 5, 7, 14, 18 and 19; a good agreement was observed for items 4, 6, 12, 13 and 17; a perfect agreement was observed for item 1.

#### Rating of key methodological items

The median MIS was 1 with a minimum of 0 and a maximum of 3. Some items’ reporting was poor, especially in the items of ‘randomization’, ‘allocation concealment and implementation’, ‘blinding’ and ‘ITT analysis’ with a positive rate of less than 10% (Table 2). A substantial agreement was observed for items 9, 10 and 11; a good agreement was observed for item 8, 15 and 16.

#### Exploratory analysis: factors associated with better reporting quality

In the univariate analysis, year of publication was the only factor associated with an increased OQS. In detail, the mean OQS increased by about 0.52 for manuscripts published in the period of 5-year increments (95% CI: 0.32 to 0.72; *P* < 0.001) (Table 3). With regard to the MIS, no variable was statistically significant in the ordinal regression model.

**Table 3:** **Simple linear regression analysis for factors associated with better overall quality of reporting rating using items from** the **Consolidated Standards for Reporting Trials (CONSORT) statement (n = 246)**

**Variables**		**SE**	***t***	***P***	**95% CI**
Constant	5.67	0.35	16.0	< 0.001	4.97 to 6.36
Year of publication	0.52	0.10	5.12	< 0.001	0.32 to 0.72

SE, standard error.

## Discussion

Our study found that the reporting quality of overall and key methodological items was not satisfactory in RCTs of melasma conducted in China. This indicated that RCTs in this field still needed improvement to meet the standard of reporting quality required by the CONSORT statement. There seemed to be an improvement in time for OQS as the mean OQS increased by about 0.52 for manuscripts published in the period of 5-year increments, which indicated that more and more scholars were realizing the importance of reporting in RCTs due to the widely adoption and promotion of CONSORT.

However, we identified some items where information was insufficient or inadequate in most studies, such as ‘randomization’, ‘allocation concealment and implementation’, ‘blinding’, ‘ITT analysis’, and so on. These key methodological items are critical in avoiding selection, performance/detection, and attrition bias. An overestimation of treatment effects has been demonstrated in trials with inadequate key methodological design comparison with trials that adequately reported these methodological items [15]. Some previous studies also found it was easy to ignore the reporting importance of some key methodological items with a positive rate of less than 30% and similar overall quality of reporting had the possibility of hiding important differences in methodological quality [7,8,10,16]. Especially, for studies in RCTs of melasma mainly conducted outside China, it is reported that there is a positive rate of 65%, 20% and 95% for items of adequate ‘randomized sequence generation’, ‘allocation concealment’ and ‘blinding’, respectively [17]. This positive rate is higher than that in our study. Other studies [16,18,19] conducted in China in other fields reported that the categories of ‘randomization’, ‘blinding’ and ‘ITT’ were used in about 15 to 30%, 0 to 20% and 0 to 10% of trials, respectively, which were similar to the results in our study. Meanwhile, ‘trial design’, ‘sample size’, ‘recruitment’ and ‘ancillary analyses’ should be emphasized in reports.

One limitation we should point out is that, due to the language barrier, we did not search for any manuscripts published in non-Chinese or English journals. This is a worthwhile area for future study. It remains unknown whether searching in other language journals would have altered the constitution of our sample or results. Despite this limitation, we think our results have good internal validity. Firstly, it is a meaningful topic to assess the quality of RCT- reporting in the treatment of melasma conducted in China as more and more RCTs in this field are emerging and a full review is lacking. Secondly, in our survey, the selection and abstraction processes were independently performed by two qualified assessors who were well-trained in mastering the CONSORT statement and have good agreement in item evaluation reporting.

## Conclusions

Our findings demonstrate that the reporting quality of RCTs in the treatment of melasma conducted in China is lacking especially in key methodological items. Reporting of RCTs in this field should meet and keep up with the standards of the CONSORT statement. It is believed that with the guidance of the CONSORT statement, more and more high quality RCT-reporting on the treatment of melasma conducted in China will emerge over time.

## Additional file

Additional file 1:
**RCTs reporting in the treatment of melasma conducted in China.**

## Abbreviations

CENTRAL: Cochrane Central Register of Controlled Trials
CONSORT: Consolidated Standards for Reporting Trials
ITT: intention-to-treat
MIS: methodological index score
OQS: overall quality score
RCT: randomized controlled trial
